# The time is now: expedited HIV differentiated service delivery during the COVID‐19 pandemic

**DOI:** 10.1002/jia2.25503

**Published:** 2020-05-01

**Authors:** Lynne Wilkinson, Anna Grimsrud

**Affiliations:** ^1^ International AIDS Society Johannesburg South Africa; ^2^ Centre for Infectious Epidemiology and Research University of Cape Town Cape Town South Africa

**Keywords:** HIV, ART, differentiated service delivery, stable, COVID‐19, pandemic, emergency response, supply chain, Dolutegravir, stock out, severe acute respiratory syndrome coronavirus 2, SARS‐CoV‐2, multi‐month dispensing

At its core, differentiated service delivery (DSD) for HIV is centred around clients’ needs and expectations and relieving unnecessary burdens on the health system [1]. In the 2016 World Health Organization (WHO) antiretroviral therapy (ART) guidelines, it was acknowledged that adaptations to the delivery of HIV services were necessary to achieve the “treat all” recommendation [2]. This transition from a “one‐size‐fits‐all approach” to DSD means modifying the location, frequency and package of services as well as the cadre providing services, considering the clinical needs, specific population and the context including urbanicity, stability of context (for example high migration, conflict or pandemic) and type of HIV epidemic [2, 3, 4]. Existing global and national policies around DSD for HIV can be leveraged during the COVID‐19 pandemic to play a critical role in supporting uninterrupted ART and reducing avoidable contact with health facilities, thereby supporting health systems to focus on COVID‐19. Recent statements from The Global Fund for HIV, Tuberculosis and Malaria (Global Fund), the Global Network of People Living with HIV (GNP+), UNAIDS, the United States President's Emergency Plan for AIDS Relief (PEPFAR) and the WHO all endorse leveraging components of DSD for people living with HIV (PLHIV) during the COVID‐19 pandemic [4, 5, 6, 7, 8, 9]. The time is now to accelerate access to DSD for all PLHIV.

We acknowledge that accelerating access requires capacity in addition to policy decisions. Scaling up the provision of longer ART refills is highly dependent on supply chains that may be currently under threat. There are concerns of constraints in ART production in the coming months as a result of the lockdown in India; with the Global Fund currently rating the operational risk assessment as “moderate” [10]. The global supply situation is being closely monitored and coordinated by partners with PEPFAR tempering language around the duration of multi‐month dispensing to ensure continuity of care [11, 12]. Countries are being encouraged to submit orders well in advance, adjust supply plans for longer leads times, distribute stock to clinics rather than holding it centrally and transparently communicate stock levels by regimen at the national and provincial level to support planning [11, 13, 14]. Concerns regarding ritonavir‐boosted lopinavir availability pre‐dated COVID‐19, and plans to transition to other antiretrovirals are being accelerated in countries with large numbers of patients on this regimen [15]. With many sub‐Saharan countries’ forecasts increasing numbers of PLHIV on a Dolutegravir (DTG)‐based first‐line regimen and a slower enrolment and transition to DTG regimens than predicted, this stock may be less threatened than the Efavirenz (EFV)‐based regimens. This further strengthens the need for supporting patients to immediately transition to DTG regimens *without* requiring additional clinical monitoring visits to health facilities.

## 1 THE PRECEDENT FOR ACCELERATING ACCESS TO DSD IN EMERGENCY CONTEXTS

There is precedent for expediting DSD approaches during times of emergency. During the 2014‐2015 Ebola outbreak, Guinea started providing PLHIV with 6‐month ART refills – both to ensure that patients were not exposed to Ebola by visiting health facilities and because many health facilities closed [16, 17]. In Sierra Leone, peers started collecting and distributing ART refills to patients’ homes or from community meeting points [18]. In response to conflict in the Central African Republic in 2015, patients were provided with 6‐month refills distributed by lay healthcare workers from decentralized peripheral health facilities [19]. More recently, in 2019 during armed conflict in the Cabo del Gado province of Mozambique, mobile clinics provided outreach and ART refills within communities [20].

## 2 LEVERAGING AND ADAPTING DSD IN RESPONSE TO THE COVID‐19 PANDEMIC

At a time when we lack data on the clinical outcomes of PLHIV co‐infected with severe acute respiratory syndrome coronavirus 2 (SARS‐CoV‐2), and in the context of resource constrained public health systems where millions of PLHIV are not on treatment or virally suppressed, we need to take every precaution possible to avoid SARS‐CoV‐2 co‐infection. DSD provides the necessary tools to take these precautions; most importantly by adapting the “when” and “where” through reducing the frequency of visits and enabling ART refills outside of health facilities [1, 21]. A number of adaptations to DSD are outlined below and should be considered in addition to implementation of critical infection control and physical distancing measures that are recommended for everyone.

## 3 EXPANDING ACCESS TO DSD AMONG PLHIV ON ART

For those clinically stable on ART but not yet enrolled in a DSD model, we must accelerate access by revising eligibility criteria and ensuring those with one suppressed viral load or evidence of treatment success after six months on treatment are immediately enrolled. Out‐of‐facility models for ART refills should be prioritized over facility‐based models. Refills should be extended to a minimum of three months with 6‐month refills permitted where stock allows; even if only as a once off or only for a specific regimen. All ART patients not yet clinically stable should receive a six‐month prescription at their next scheduled appointment and a minimum 3‐month treatment supply to ensure the most vulnerable PLHIV reduce health facility visits unless unwell. Longer prescriptions will allow for flexibility should it not be appropriate for patients to return to a health facility after three months.

## 4 ADAPTING DSD FOR THOSE CURRENTLY RECEIVING THEIR ART THROUGH A DSD MODEL

For those already in a DSD model, the priorities should be to further reduce interactions with health facilities and align with the WHO HIV‐COVID‐19 recommendations that all PLHIV have a minimum of 30 days of ART with them; but preferably a supply of three to six months [2, 22, 23]. Where the DSD model takes place in a facility, such as through fast track and facility adherence clubs, infection control and physical distancing measures should be urgently put in place (e.g. triaging PLHIV with COVID‐19 symptoms and providing their refills in separate area to other PLHIV), relocating refill collection to outside the facility buildings, advising and managing PLHIV queuing at least a metre and a half apart while waiting, and collecting treatment individually with no facilitated group interactions). A core priority must be to ensure that PLHIV can leave the facility or community venue after the shortest possible time, ideally with a single point of contact. As has already been recommended by the ministries of health in sub‐Saharan Africa, community‐based group DSD models should transition from meeting in‐person to staying connected via telephone or vitually, if possible [24].

## 5 REACHING PLHIV NOT ON ART

In the context of COVID‐19, WHO highlights their 2017 recommendation that PLHIV not on ART should immediately start ART [25]. For PLHIV not yet on ART, informing them about the importance of taking ART to strengthen their immune system is now more critical than ever given that immunosuppression of HIV could place them at greater risk for COVID‐19 [9]. PLHIV without COVID‐19 symptoms should be started on ART on the day of diagnosis, preferably on a DTG‐regimen [26], at the location of the diagnosis and provided a 3‐month supply [12] at initiation to reduce the need to visit a health facility during COVID‐19, with greater emphasis placed on initiation outside of facilities (e.g. through outreach and mobile services).

In conclusion, if ever there was a time to provide extended ART refills, and offer them outside of conventional healthcare facilities, now is the time. We call on health services and supporting partners to expedite the implementation of DSD to empower and protect PLHIV and capacitate health systems to respond to the COVID‐19 pandemic.

## COMPETING INTERESTS

None of the authors have competing interests to declare.

## AUTHORS’ CONTRIBUTIONS

The concept for this commentary was developed by LW and AG. LW wrote the first draft. All authors contributed and approved the final version.

## References

[1] Grimsrud A , Bygrave H , Doherty M , Ehrenkranz P , Ellman T , Ferris R , et al. Reimagining HIV service delivery: the role of differentiated care from prevention to suppression. J Int AIDS Soc. 2016;19(1):21484. 27914186 10.7448/IAS.19.1.21484 PMC5136137

[2] World Health Organization . Consolidated guidelines on the use of antiretroviral drugs for treating and preventing HIV infection. Recommendations for a Public Health Approach. Geneva, Switzerland; 2016. 27466667

[3] Duncombe C , Rosenblum S , Hellmann N , Holmes C , Wilkinson L , Biot M , et al. Reframing HIV care: putting people at the centre of antiretroviral delivery. Trop Med Int Health. 2015;20(4):430–47. 25583302 10.1111/tmi.12460 PMC4670701

[4] Gustav R .Lessons learnt from the HIV response for COVID‐10: Building community resilience. 2020 [cited 2020 Mar 23]. Available from: https://www.gnpplus.net/lessons-learnt-from-the-hiv-response-for-covid-19-building-community-resilience/

[5] The Global Fund to Fight HIV, TB and Malaria . COVID‐19 information note: considerations for global fund HIV support. 2020 [2020 Apr 9]. Available from: https://www.theglobalfund.org/media/9512/covid19_hiv_infonote_en.pdf

[6] UNAIDS . Rights in the time of COVID‐19: lessons from HIV for an effective, community‐led response. 2020 [cited 2020 Mar 23]. Available from: https://www.unaids.org/sites/default/files/media_asset/human-rights-and-covid-19_en.pdf

[7] UNAIDS . Successful global epidemic responses put people at the centre. 2020 [cited 2020 Mar 23]. Available from: https://www.unaids.org/en/resources/presscentre/featurestories/2020/march/20200312_covid-19

[8] Office of the Global AIDS Coordinator . PEPFAR technical guidance in context of COVID‐19 pandemic. Washington, DC: PEPFAR; 2020.

[9] World Health Organization . Q&A on COVID‐19, HIV and antiretrovirals. 2020 [cited 2020 Mar 23]. Available at: https://www.who.int/news-room/q-a-detail/q-a-on-covid-19-hiv-and-antiretrovirals

[10] The Global Fund to Fight HIV, TB and Malaria . COVID‐19 impact on health product supply: assessment and recommendations. 2020 [cited 2020 Apr 9]. Available from: https://www.theglobalfund.org/media/9440/psm_covid-19impactonsupplychainlogistics_report_en.pdf

[11] Badiane K .HIV drug distribution: Increasing patient‐centered care and minimizing PLHIV exposure to COVID‐19. Presentation during “Differentiated service delivery and COVID‐19 webinar”. 2020 [cited 2020 Apr 9]. Available from: https://cquin.icap.columbia.edu/news/webinar-series-differentiated-service-delivery-dsd-and-the-covid-19-response/

[12] PEPFAR . PEPFAR technical guidance in context of COVID‐19 pandemic. 2020.

[13] The Global Fund to Fight HIV, TB and Malaria . Global fund supports countries in response to COVID‐19. 2020 [cited 2020 Apr 9]. https://www.theglobalfund.org/en/updates/other-updates/2020-03-23-global-fund-supports-countries-in-response-to-covid-19/

[14] Ngugi C .DSD in the context of COVID‐19: Kenya situation. Presentation during “Differentiated service delivery and COVID‐19 webinar”. 2020 [cited 2020 Apr 2]. Available from: https://cquin.icap.columbia.edu/wp-content/uploads/2020/04/CQUIN-COVID-webinar_March-31.pdf

[15] ARV Procurement Working Group . LPVr 100_25mg capacity constraint contingency plan. 2020 [cited 2020 Apr 9]. Available from: https://www.arvprocurementworkinggroup.org/arv-procurement-working-group-documents

[16] Beklolo CE , Diallo A , Philips M , Yuma J‐D , Di Stefano L , Drèze S , et al. Six‐monthly appointment spacing for clinical visits as a model for retention in HIV care in Conakry‐Guinea: a cohort study. BMC Infect Dis. 2017;17:766. 29237401 10.1186/s12879-017-2826-6 PMC5729484

[17] International AIDS Society . Prioritizing differentiated ART delivery for clinically stable clients in West and Central Africa. 2019. [cited 2020 Apr 09]. Available from: http://www.differentiatedcare.org/Portals/0/adam/Content/unwjL‐Yg_ECyAiBEEWeqPw/File/12%20pager%20english%20WEB.pdf

[18] Ellie M , Massaquoi S .Demand driven DSD policy development. The experience of Sierra Leone. Presentation from IAS Consultation on Differentiated ART Delivery in West and Central Africa. Accra: Ghana. 2019. http://differentiatedservicedelivery.org/Portals/0/adam/Content/B9_ieYV76UOOW-LB9YHSEg/File/3_DSD%2520policy%2520Sierra%2520Leone_Martin%2520Ellie%2520and%2520Samuel%2520Massaquoi.ppt

[19] Ssonko C , Gonazlez L , Mesic A , da Fonseca MS , Achar J , Safar N , et al. Delivering HIV care in challenging operating environments: the MSF experience towards differentiated models of care for settings with multiple basic health care needs. J Int AIDS Soc. 2017, 20 Suppl 4: 21654. 28770590 10.7448/IAS.20.5.21654 PMC5577706

[20] PEPFAR Mozambique .Session 6B_Care Treatment_2020.02.26_FINAL. Presentation during PEPFAR COP 20 Planning Meetings; Johannesburg, South Africa. 2020.

[21] International AIDS Society (IAS) . Differentiated care for HIV: A decision framework for antiretroviral therapy. Durban, South Africa; IAS 2016 [cited Mar 23]. Available from: http://differentiatedservicedelivery.org/Portals/0/adam/Content/yS6M-GKB5EWs_uTBHk1C1Q/File/Decision%2520Framework%2520Version%25202%25202017.pdf

[22] WHO Questions and Answers on COVID‐19, HIV and anti‐retrovirals. 2020 [cited 2020 Apr 09]. Available from: https://www.who.int/news-room/q-a-detail/q-a-on-covid-19-hiv-and-antiretrovirals

[23] WHO, CDC, PEPFAR, USAID, IAS . Key considerations for differentiated antiretroviral therapy delivery for specific populations: Children, adolescents, pregnant and breastfeeding women and key populations. 2017 [cited 2020 Apr 2]. Available from: https://www.who.int/hiv/pub/arv/hiv-differentiated-care-models-key-populations/en/

[24] Moçambique Ministério da saúde . Assunto: Pacote de Serviços para Populações vivendo com o HIV no âmbito da resposta ao COVID – 19. 2020.

[25] World Health Organization . Guidelines for managing advanced HIV disease and rapid initiation of antiretroviral therapy. Geneva: Switzerland: World Health Organization; 2017. 29341560

[26] World Health Organization . Update of recommendations on first‐ and second‐line antiretroviral regimens. Geneva: Switzerland: World Health Organization; 2019.

